# Achieving ultranarrow graphene perfect absorbers by exciting guided-mode resonance of one-dimensional photonic crystals

**DOI:** 10.1038/srep32312

**Published:** 2016-08-31

**Authors:** Yongbing Long, Liang Shen, Haitao Xu, Haidong Deng, Yuanxing Li

**Affiliations:** 1 College of Electronic Engineering, South China Agricultural University, Guangzhou, 510642, China; 2 State Key Laboratory on Integrated Optoelectronics, College of Electronic Science and Engineering, Jilin University, 2699 Qianjin Street, Changchun 130012, China; 3 School of Applied Physics and Material, WuYi University, Jiangmen, 529020, China

## Abstract

Graphene perfect absorbers with ultranarrow bandwidth are numerically proposed by employing a subwavelength dielectric grating to excite the guided-mode resonance of one-dimensional photonic crystals (1DPCs). Critical coupling of the guided-mode resonance of 1DPCs to graphene can produce perfect absorption with a ultranarrow bandwidth of 0.03 nm. The quality factor of the absorption peak reaches a ultrahigh value of 20000. It is also found that the resonant absorption peaks can be tuned by controlling the dispersion line of the guided mode and the period of the grating. When the parameters of the grating and the 1DPCs are suitably set, the perfect absorption peaks can be tuned to any randomly chosen wavelength in the visible wavelength range.

Graphene, a one-atom thick and two-dimensional material, has attracted increasing attention due to its remarkable electronic, mechanical and optical properties^1^,^2^. This material also has great potential to be applied to optoelectronic devices such as photodetectors, biosensing, optical modulators, and photovoltaic cells^1^,^2^,^3^,^4^,^5^,^6^,^7^,^8^,^9^,^10^. However, in the visible and near-infrared range, graphene only absorbs about 2.3% of the light at normal incidence, which is a severe limitation to its further application in optoelectronic devices. Tremendous research efforts have been made to improve light absorption in graphene and various methods have been presented to enhance the light-graphene interaction by improving the optical electric field around the graphene^11^,^12^,^13^,^14^,^15^,^16^,^17^,^18^,^19^,^20^,^21^,^22^,^23^,^24^,^25^,^26^,^27^,^28^. For example, Fabry-Perot microcavity is constructed by integrating graphene between two mirrors^16^,^17^. The microcavity can confine large optical electric field when it resonates and then significantly improve light absorption in graphene; but the enhanced optical absorption cannot be conveniently used for application in biosensing where graphene is required to interact with the surrounding environment. In other studies, plasmonic nanostructures such as metal nanogratings and nanoparticles have been constructed for the same purpose, but light absorption is still low and the bandwidth of the absorption peaks is broad due to the optical loss of the metal^18^,^19^,^20^,^21^,^22^,^23^,^24^.

More recently, hole-array photonic crystal slab and slit-array dielectric grating are employed to enhance graphene absorption^25^,^26^,^27^. The hole-array/slit-array can diffract the incident light into evanescent electromagnetic waves and then excite the guided-mode resonance within the photonic crystal slab/dielectric grating. Critical coupling of these guided-mode resonances to graphene can improve light absorption in a single graphene or graphene strips up to 100%. The hole-array photonic crystal slab and the slit-array dielectric grating, therefore, are considered a good option to achieve perfect absorption peaks within the visible and near-infrared region. The bandwidth of the perfect absorption peaks, however, remains above several nanometers and the quality factor (Q-factor) of these absorption peaks is still relatively low (only several hundreds).

This paper aims to propose a new type of graphene perfect absorbers (GPAs) with ultrahigh Q-factor and ultranarrow bandwidth by exciting the guided-mode resonance of one-dimensional photonic crystals (1DPCs). For this purpose, a subwavelength dielectric grating is capped on the 1DPCs to excite the guided-mode resonances. As 1DPCs are multilayers with low and high refractive index distributed periodically, the guided mode of this type of multilayers is then modulated by the periodic distribution of refractive index^29^,^30^,^31^. When this type of guided mode of 1DPCs is excited by the grating, it is dually modulated by the periodic distribution of refractive index in the 1DPCs and the periodicity of the dielectric grating. Critical coupling of this dually modulated guided-mode resonance to graphene produces perfect absorption with a ultranarrow bandwidth of 0.03 nm, a value about two orders of magnitude lower than that of the GPAs presented in previous research^25^,^26^,^27^. Correspondingly, the Q-factor of the absorption peak reaches a much higher value of 20000.

## Results and Discussions

### Achieving GPAs by exciting dually modulated guided-mode resonance of 1DPCs

**Figure 1:**
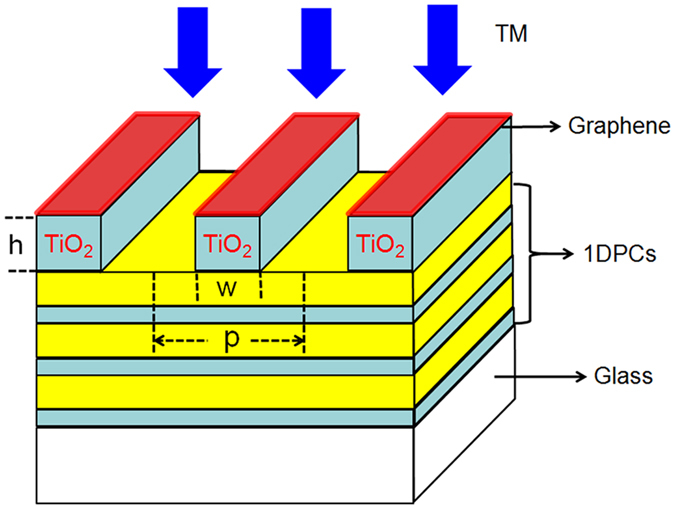
Structure of the device used to achieve perfect absorption. p, w and h respectively denote period, strip width and thickness of the subwavelength grating.

The proposed GPAs have a structure of graphene strip array/subwavelength TiO_2_ grating/1DPCs, as is shown in Fig. 1(a). The 1DPCs are assumed to be composed of 10 pairs of (SiO_2_/TiO_2_) and the subwavelength TiO_2_ grating is placed on 1DPCs to excite the guided mode of the 1DPCs. The thickness, period and strip width of the subwavelength gratings are h, p and w, respectively. Finite element method is employed to calculate the optical electric field and the light absorption spectrum for the graphene^32^,^33^. More details concerning the calculation can be found in the part Method. In the calculation, the refractive index of TiO_2_ and SiO_2_ are assumed to be 2.5 and 1.5. The graphene is assumed to be 0.34 nm and the refractive index is estimated to be *n* = 3.0 + *iC*_1_*λ*/3 with *C*_1_ = 5.446 *μm*^−1 ^34.

To obtain GPAs, light absorption in graphene strips is optimized by tailoring the thickness, period and strip width of the grating. For this purpose, Monte Carlo method is first used to calculate light absorption in graphene strips by randomly selecting the parameters (h, p and w) of the grating in a broad range. With this method, a series of parameters are obtained for the devices within which light absorption in graphene shows relatively high values. These parameters are then used as initial values to optimize the device structure by calculating light absorption of graphene as a function of h, p and w.

**Figure 2:**
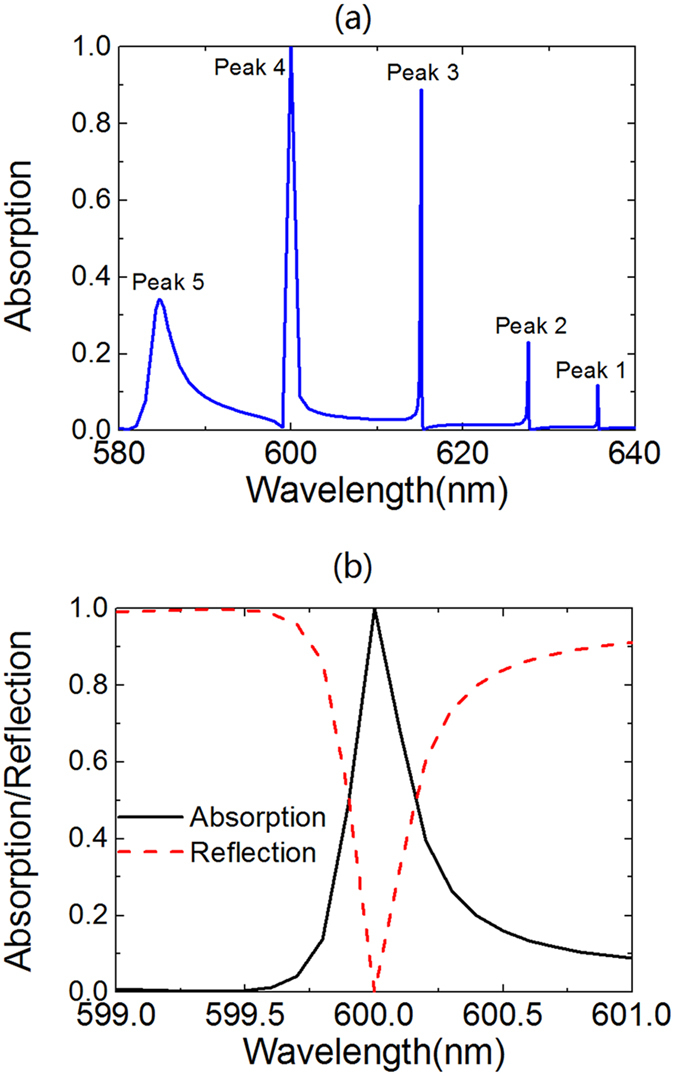
(**a**) Absorption spectrum of graphene strips in Device I; (**b**) Amplification of the absorption spectrum around 600 nm. The red dashed line in (**b**) denotes the reflection spectrum of Device I.

In the calculation, the wavelength of the incident light is randomly set as 600 nm and the polarization is assumed to be transverse magnetic. The center wavelength (λ_0_) of the photonic bandgap of 1DPCs is set as 600 nm and the reflectance of 1DPCs is 100% at this wavelength. Correspondingly, the thickness of TiO_2_ and SiO_2_ layers in 1DPCs is calculated by λ_0_/4n with n as the refractive index of TiO_2_ or SiO_2_. After careful optimization, light absorption in the graphene strips is maximized when w, p and h of the grating are respectively set as 365.0 nm, 197.4 nm and 124.1 nm. The device with these parameters is named as Device I and the calculated absorption spectrum of the device is shown in Fig. 2. From the figure, two main points can be observed. First, the absorption spectrum shows five peaks at the wavelength of 635.7 nm, 627.6 nm, 615.1 nm, 600 nm and 584.7 nm. These peaks are named as Peak *i* (*i* = 1, 2, 3, 4, 5), as is shown in Fig. 2(a). Second, absorption at the wavelength of 600 nm (Peak 4) reaches 100%, indicating perfect absorption in the graphene strips is achieved (see Fig. 2(b)).

**Figure 3:**
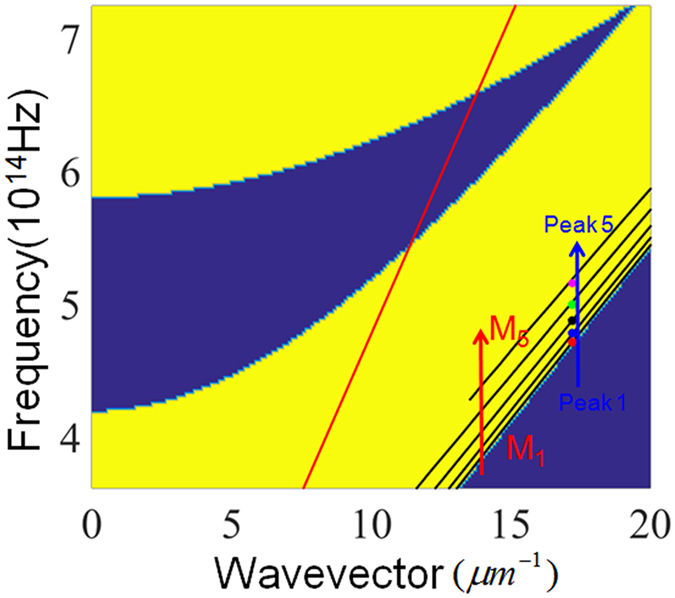
Dispersion diagram of 1DPCs. The line M*i* (*i* = 1, 2, 3, 4, 5) denotes the *i*th-order dispersion line of the guided mode of 1DPCs. Points with different colors correspond to Peak *i* on the absorption spectrum of Device I. The red line denotes the light curve.

**Figure 4:**
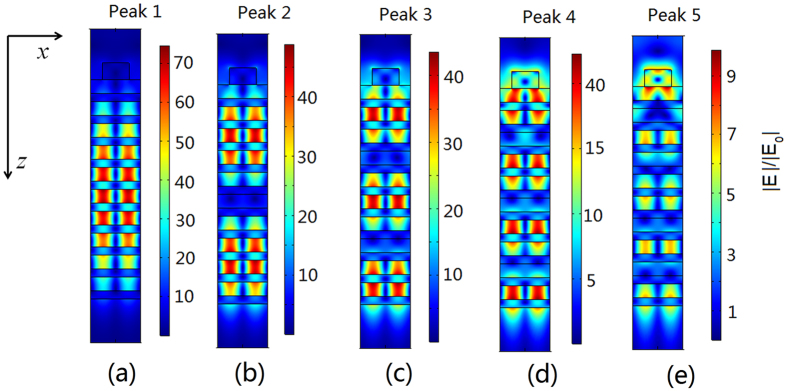
Distribution of the optical electric field in Device I when the wavelength of the incident light is positioned at absorption Peak *i* (*i* = 1, 2, 3, 4, 5). (**a**): for Peak 1; (**b**): for Peak 2; (**c**): for Peak 3; (**d**): for Peak 4; (**e**): for Peak 5. |E| is the modulus of optical electric field in the device and |E_0_| denotes that of the incident light.

The multi-peaking absorption spectrum and the perfect absorption in graphene strips are attributed to the excitation of the guided-mode resonance of the 1DPCs. This is examined by calculating the dispersion diagram of 1DPCs and the dispersion line of the guided mode of 1DPCs^29^,^30^,^31^. The dispersion line of the guided mode is determined by using a prism to excite the guided mode of 1DPCs and more details can be found in Supplementary Information. The calculated results are shown in Fig. 3, where line M*i* (*i* = *1*, *2*, *3*, *4*, *5*) denotes the first-order to the fifth-order guided mode of the 1DPCs. To reveal the relation between the guided mode and the absorption peaks, we superpose the reciprocal lattice vector of the grating (g) and frequency of the absorption peaks (*f*_*i*_, *i* = *1*, *2*, *3*, *4*, *5*) onto the dispersion diagram. It is found that the points (g, *f*_*i*_) related to the absorption Peak *i* are well matched with the dispersion line M*i* of the guided mode. This indicates the excitation condition of the guided mode is fulfilled^29^,^30^,^31^:


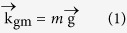


where 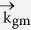 denotes the wavevector of the guided mode of the 1DPCs; 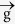 denotes the reciprocal lattice vector of the grating and g is calculated by g = 2π/p where p is the period of the grating; m is the grating diffraction order and m is equal to 1 in this paper. The matching of Eq. (1) allows the excitation of the guided mode of the 1DPCs, which in turn enhances light absorption in the graphene strips and leads to the peaks on the absorption spectrum. More specifically, Peak 1 to 5 on the absorption spectrum are caused by the first-order to the fifth-order guided mode of the 1DPCs. The judgment is further confirmed by calculating distribution of the optical electric field in Device I and the calculated results are shown in Fig. 4. It is observed that the envelope of the optical electric field shows *i* (*i* = *1*, *2*, *3*, *4*, *5*) maxima at the direction parallel (*z*-direction) to the incident light. These maxima result from the standing-wave modes in the waveguide created by the 1DPCs^29^. This finding verifies that the multi-peaking on the absorption spectrum of Device I results from the guided-mode resonance of the 1DPCs.

Figure 4 also demonstrates that the optical electric field oscillates at both the parallel (*z*-direction) and perpendicular (*x*-direction) direction to the incident light. The oscillating behavior at *z*-direction is attributed to the periodic modulation of the refractive index in the 1DPCs and at *x*-direction it is caused by the periodicity of the dielectric grating. Due to the dual modulation, the guided mode of 1DPCs resonates at both the parallel (*z*-direction) and perpendicular (*x*-direction) direction to the incident light, which significantly improves the optical electric field inside the 1DPCs. The dually modulated guided-mode resonance of the 1DPCs can leak evanescent waves to the interface of grating/air and subsequently improve the light graphene interaction, as is shown in Fig. 4. When the external leakage energy of the guided-mode resonance is equal to the intrinsic loss of graphene strips (i.e., the condition for critical coupling is fulfilled), critical coupling occurs and total absorption of the light is achieved at the wavelength of 600 nm^25^,^26^,^27^.

### Tuning the perfect absorption peaks within visible wavelength range

**Figure 5:**
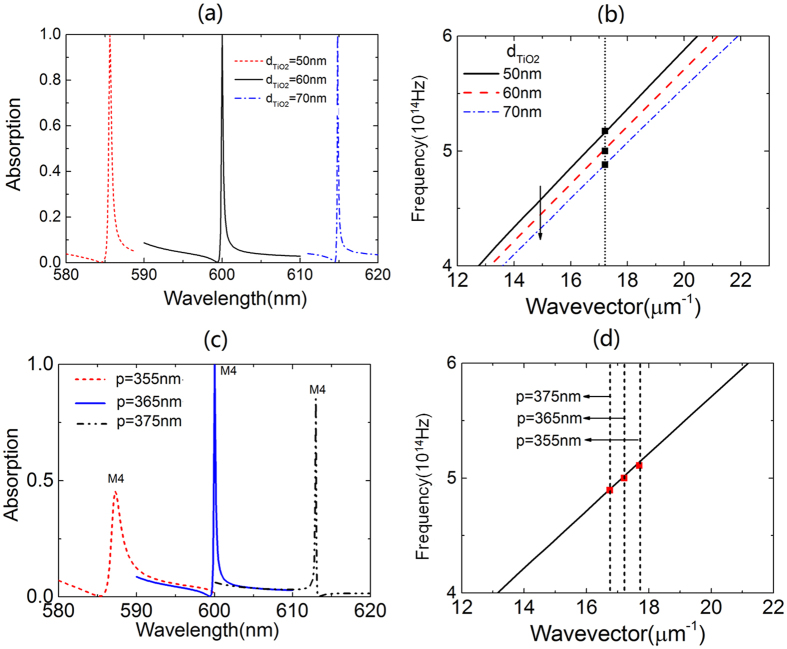
(**a**) Absorption spectra for Device I with varied thickness of the TiO_2_ layer 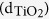 in the 1DPCs; (**b**) dispersion line of the 4th-order guided mode of 1DPCs with varied 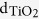 and the squares denote the absorption peaks when 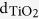 is set as varied values. (**c**) Absorption spectra for Device I with varied period (p) of the grating. (**d**) dispersion line of the 4th-order guided mode of 1DPCs with 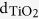 set as 60 nm and the squares denote the absorption peaks in graphene strips when period (p) of the grating is set as varied values. The dotted line in (**b**) and dashed line in (**d**) denote the reciprocal lattice vector of the grating.

The calculation above demonstrates that absorption peaks appear when the excitation condition of the guided mode is satisfied. It also suggests that the spectral position of the absorption peaks can be tuned by controlling the dispersion line of the guided mode and the period of the grating, as is indicated by Eq. (1). The judgment is examined by calculating the absorption spectrum of Device I with varied structure parameters of 1DPCs and grating. The calculated results are shown in Fig. 5.

In Fig. 5(a), the spectral position of the absorption peaks is redshifted by a value of 14.8 nm when the TiO_2_ layer in 1DPCs is increased by 10 nm. This happens because an increase in the thickness of the TiO_2_ layer can shift the dispersion line of the guided mode to the lower frequency region (see Fig. 5(b)); therefore a lower frequency (i.e., a larger wavelength) is required to match the excitation condition of the guided mode of the 1DPCs. Correspondingly, the absorption peaks are shifted to the longer wavelengths. In Fig. 5(c), an increase of 10 nm in the period of the grating leads to a redshift of 6.2 nm for the absorption peaks. This is because a grating with larger period can excite the guided mode in the longer wavelength range (i.e., guided mode with lower frequency), as is shown in Fig. 5(d). It is also observed in Fig. 5(b,d) that the points (k, *f*) related to the absorption peaks for the devices with varied thickness of TiO_2_ layer and period of grating are just positioned on the dispersion lines of the guided mode of the 1DPCs. The finding confirms the conclusion that absorption peaks occur when the excitation condition of the guided mode (i.e., Equation (1)) is matched.

**Figure 6:**
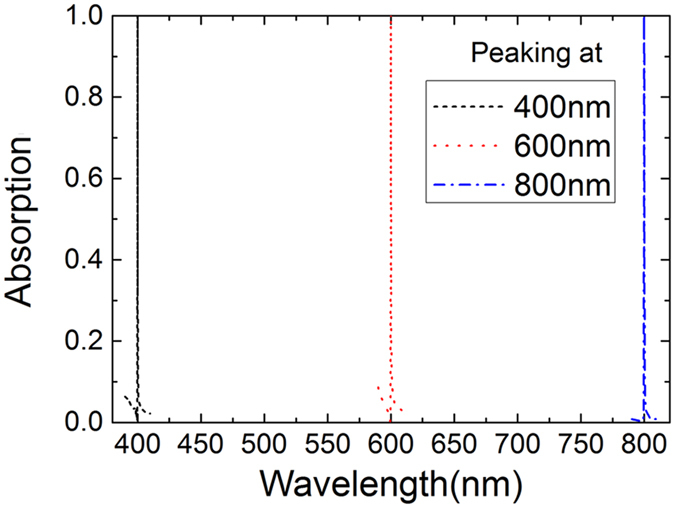
Absorption spectra for the device with perfect absorption at 400 nm, 600 nm and 800 nm.

These results demonstrate that absorption peaks are sensitive to the structure parameters of the grating and 1DPCs, and the spectral position of absorption peaks can be tuned by tailoring these parameters. In fact, when the period (p), strip width (w) and thickness (h) of the grating and the parameters of the 1DPCs are properly designed to fulfill the condition for critical coupling between the guided-mode resonance and graphene, perfect absorption can be achieved at any randomly given wavelength within the visible wavelength range. In Fig. 6, for example, perfect absorption occurs at the wavelength of 400 nm when p, w and h are respectively set as 266.3 nm, 107.8 nm and 91.3 nm. When these parameters are tailored to the values of 528.3 nm, 277.8 nm and 157.6 nm, perfect absorption is tuned to the wavelength of 800 nm. In both cases, the thickness of TiO_2_ and SiO_2_ layers in 1DPCs is determined by λ_0_/4n with λ_0_ as the wavelength of the absorption peaks and n as the refractive index of TiO_2_ or SiO_2_. In these calculations, however, refractive index of TiO_2_ and SiO_2_ is set as constant values when the wavelength varies from 400 nm to 800 nm. In practical design of GPAs, the realistic material dispersion of the refractive index should be taken into consideration.

### Achieving GPA with ultranarrow bandwidth

**Figure 7:**
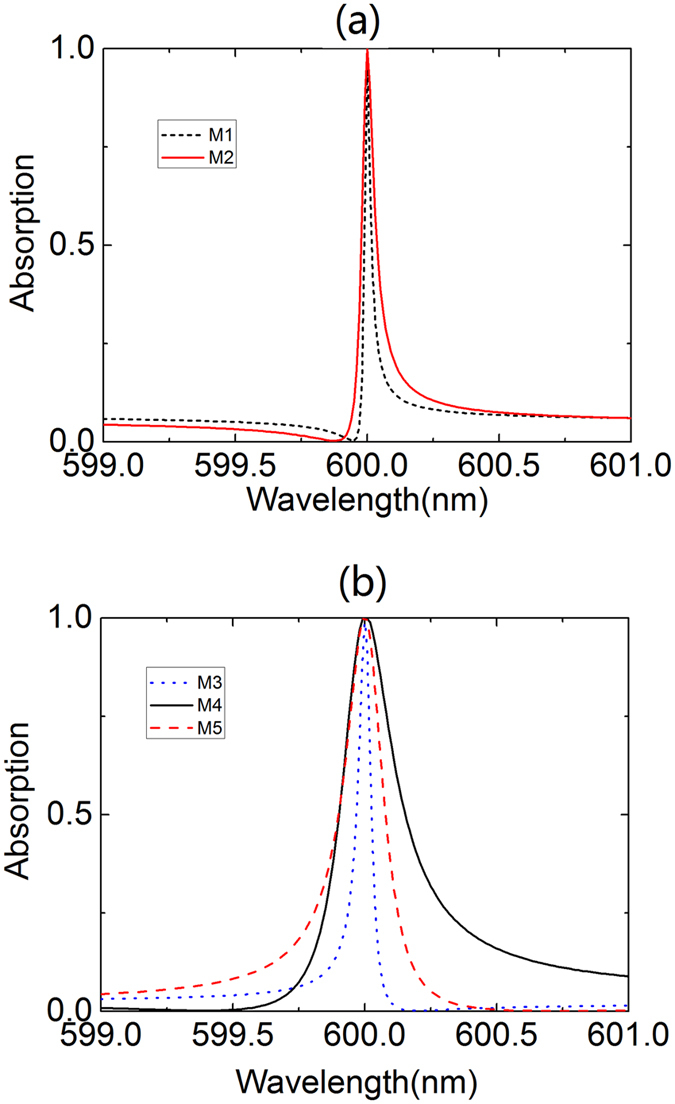
Absorption spectra for the device when different orders of guided mode are used to achieve perfect absorption at 600 nm. M*i* (*i* = 1, 2, 3, 4, 5) denotes the absorption spectrum related to the *i*th-order of the guided-mode resonance.

It is observed in Fig. 2 that absorption peaks related to lower-order guided-mode resonance has narrower bandwidth. In other words, GPAs with narrow bandwidth can be achieved by exciting low orders of the guided-mode resonance of 1DPCs. This is investigated by designing GPAs of which different orders of guided-mode resonance is excited. In the designing process, the wavelength of the incident light is assumed to be 600 nm. The center wavelength of the photonic bandgap of 1DPCs is set as 600 nm, and the thickness of TiO_2_ and SiO_2_ layers in 1DPCs is calculated to be 60 nm and 100 nm, respectively. The period of the grating is set as the values which can excite the different orders of the guided mode of the 1DPCs and these values are obtained by Eq. (1). The thickness and strip width of the grating are tailored to fulfill the condition for critical coupling between the guided-mode resonance and graphene. After optimization, it is found that perfect absorption is achieved for all orders of the guided-mode resonances (*i* = 1, 2, 3, 4, 5), as is shown in Fig. 7.

Another important result observed in Fig. 7 is that the bandwidth of the absorption peak related to the first-order guided-mode resonance is as small as 0.03 nn, a value about two order of magnitude narrower than that of the GPAs reported in relevant research so far^25^,^26^,^27^. Due to the small bandwidth, the Q-factor of the resonant peak is calculated to be as high as 20000, about two orders of magnitude higher than that of the GPAs in previous studies. Here the Q-factor is defined by Q = λ/Δλ where λ is the center wavelength of the peak and Δλ represents the bandwidth of the absorption peak. The narrower resonance is achieved because GPAs in this paper result from the excitation of the guide-mode resonance of the 1DPCs while GPAs in previous studies result from the excitation of the guided-mode resonance of either the grating or photonic crystal slab^25^,^26^,^27^. For the latter, the graphene is just positioned neighbouring to the grating or the photonic crystal slab, where guided-mode resonance significantly improves the optical electric field. The leakage rate of the energy from the guided-mode resonance to the graphene is relatively large^25^. Thus, a guided-mode resonance with low Q-factor (i.e., with less energy stored in 1DPCs) is required to achieve critical coupling and the bandwidth of the absorption peak is rendered relatively large.

But for the GPAs discussed in this paper, graphene is placed far away from the 1DPCs, where large optical electric field is confined by the guide-mode resonance (see Fig. 5). The leakage rate of the energy from the guided-mode resonance to the graphene is relatively low and resonance with high Q-factor (i.e., with more energy stored in 1DPCs) is required to fulfill the critical coupling condition. Hence, narrower absorption peaks can be achieved.

**Figure 8:**
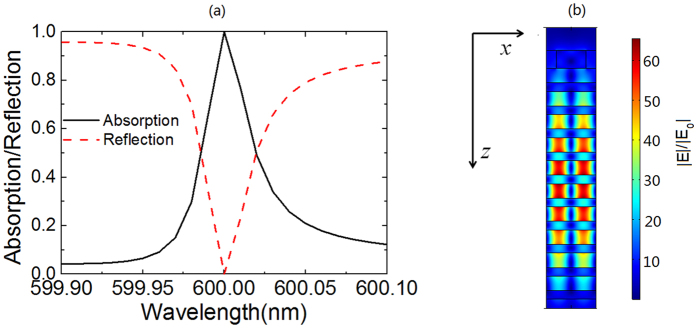
(**a**) Absorption spectrum of the infinitely extended graphene layer in Device II. The red dashed line in denotes the reflection spectrum of Device I. (**b**) Distribution of the optical electric field in Device II. |E| is the modulus of optical electric field in the device and |E_0_| denotes that of the incident light.

Interestingly, similar results can be also obtained when graphene strips are replaced by an infinitely extended graphene layer. This type of device with infinitely extended graphene on the top of grating backed by 1DPCs is referred to as Device II. Figure 8(a) demonstrates that perfect absorption is achieved for Device II when p,w and h of the grating are respectively set as 343.5 nm, 203.6 nm, 125.0 nm. The total absorption is attributed to the excitation of the first-order guided-mode resonance of 1DPCs. This can be examined by calculating the optical electric field in Device II and the calculated results are shown in Fig. 8(b). It is observed that large optical electric field is confined within the 1DPCs and the envelope of the optical electric field shows one maximum at the direction parallel (*z*-direction) to the incident light. Figure 8(b) also shows that the guided-mode resonance of 1DPCs leaks evanescent waves to graphene and then improves the light-graphene interaction, which ultimately results in the perfect absorption in the infinitely extended graphene layer.

So far, the calculation in this paper has demonstrated that perfect absorption with ultranarrow bandwidth can be achieved at a target wavelength by properly designing the device parameters. This, however, requires sub-nm fabrication precision. Considering current accuracy of fabricating techniques, the practical size of the grating will deviate from the values of the designed parameters to achieve perfect absorption at a target wavelength. As a result, the absorption peaks may be broadened and shifted away from the target wavelength, which renders it very challenging to fabricate the GPAs with ultranarrow absorption peaks at the designated target wavelength. Despite this, the deviation of the absorption peak can be restricted in a controlled wavelength region when the fabricating accuracy is included in the simulation. The physical laws revealed in this paper, we believe, shall shed new lights on finding effective approaches to achieve ultranarrow absorption peaks.

## Conclusions

GPAs with ultranarrow bandwidth are presented by using a subwavelength dielectric grating to excite the guided-mode resonance of 1DPCs. By carefully designing the structure parameters of the gratings and the 1DPCs, perfect absorption peaks can be tuned to any randomly chosen wavelength in the visible wavelength range. The bandwidth of the perfect absorption peak reaches a ultrasmall value of 0.03 nm, about two orders of magnitude higher than that of the GPAs reported in previous research. Correspondingly, the Q-factor of the absorption peak reaches a ultrahigh value of 20000. The resonant absorption peak with ultranarrow bandwidth can be considered for application in biosensing and light detection with ultra-high sensitivity.

## Method

Optical electric field in the devices is first calculated by employing finite element method^29^,^30^. In the calculation, the left and right boundaries of the computational domain are set as periodic boundary conditions; the top and bottom of the computational domain are set as absorbing boundary condition: perfectly matched layers, which are ten wavelengths away from the device. Adaptive triangle meshing with a maximum element size of 0.08 nm has been used in graphene domain. In the grating domain, the maximum element size is set as 1 nm and the minimum element size is set as 0.1 nm. With the optical electric field obtained from finite element method as input parameters, light absorption in graphene is then calculated as 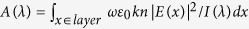, where ε_0_ is the permittivity of vacuum, λ and I(λ) are respectively the wavelength and intensity of the incident light; n and k denote the refraction index and extinction coefficient of graphene; E(*x*) is the electrical optical field in graphene^32^,^33^,^35^.

The dispersion line of the guided mode of 1DPCs is determined by the method presented in the supporting information. In this method, a prism is attached to the 1DPCs and reflectance is calculated as a function of the incident angle. The reflectance shows dips when the angle increases. With the incident angle of these dips as input parameters, the wavevector of the guided mode can be calculated and the mode dispersion relations can be obtained. More details can be found in the Supplementary Information.

## Additional Information

**How to cite this article**: Long, Y. *et al*. Achieving ultranarrow graphene perfect absorbers by exciting guided-mode resonance of one-dimensional photonic crystals. *Sci. Rep.* **6**, 32312; doi: 10.1038/srep32312 (2016).

## Supplementary Material

Supplementary Information
