# Supplementary material for: Achieving ultranarrow graphene perfect absorbers by exciting guided-mode resonance of one-dimensional photonic crystals
Source: Sci Rep. 2016 Aug 31;6:32312. doi: 10.1038/srep32312 (PMC5006081; doi:10.1038/srep32312)
Supplement: Supplementary Information [file srep32312-s1.pdf]

Supplementary Information

Achieving ultranarrow graphene perfect absorbers by exciting guided-mode resonance of one-dimensional photonic crystals

Yongbing long[1*], Liang Shen[2], Haitao Xu[1], Haidong Deng[1], Yuanxing Li[3]

1College of Electronic Engineering, South China Agricultural University, Guangzhou, 510642,China

2 State Key Laboratory on Integrated Optoelectronics, College of Electronic Science and Engineering, Jilin University, 2699 Qianjin Street, Changchun 130012, China

3 School of Applied Physics and Material, Wu Yi University, Jiangmen, 529020, China

[*]yongbinglong@126.com

To determine the dispersion relation of the guided mode of 1DPCs, a prism is used to excite the guided mode. As is shown in Fig. S1, a prism is attached to the 1DPCs with a structure of $(TiO_2/SiO_2)^{10}$. Transferred matrix method is employed to calculate the reflectance as a function of the incident angle. In the calculation, the frequency (*f*) of incident light is assumed to be $5 \times 10^{14} Hz$ (i.e., the wavelength is 600nm).[S1] The refractive index of TiO_2 and SiO_2 in the 1DPCs are assumed be 2.5and 1.5, respectively. To identify the guided mode, the TiO_2 and SiO_2 layer are assumed to have an extinction coefficient of 10^{-3}. The calculated results are shown in Fig. S2. It is observed that the reflectance shows dips when the incident angle increases. These reflectance dips indicate that the guided mode of 1DPCs is excited. The excitation of the guided mode is confirmed by calculating the optical electric field in 1DPCs (see Fig. S3). The figure demonstrates that the optical electric field in the 1DPCs shows *i* maxima for the reflectance Dip *i*. This finding verifies the conclusion that the *i*th-order guided mode of 1DPCs is excited at the reflectance Dip *i*.

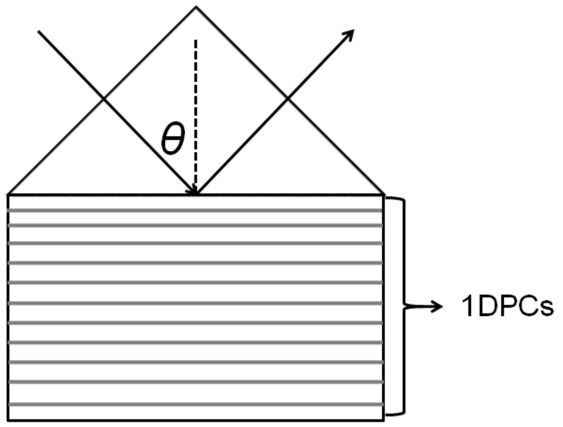

Figure S1 Structure of the device where a prism is used to excite the guided mode of 1DPCs

The excitation condition of the guided mode of 1DPCs can be expressed as[S2-S4]:

$$\vec{k}_{gm} = \overrightarrow{k_p} \sin\theta_{dip} \qquad \text{(S1)}$$

where $\vec{k}_{gm}$ denotes the wavevector of the guided mode of the 1DPCs; $\vec{k}_p$ denotes the wavevector of the incident light in the prism; k_p is calculated as $2\pi nf/c$ where n, f and c are the refractive index of the prism, frequency of the incident light and velocity of the incident light in vacuum, respectively; θ_{dip} is the incident angle where the reflectance shows dips.

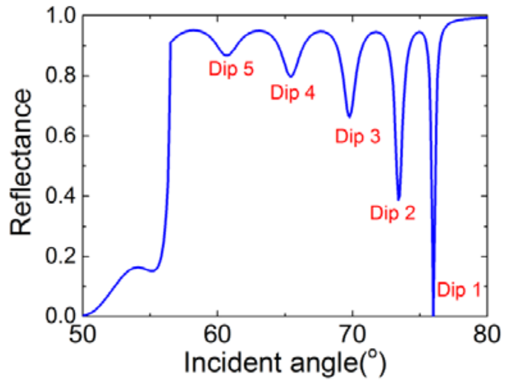

Figure S2 Angular reflectance spectrum at frequency of $5 \times 10^{14} Hz$

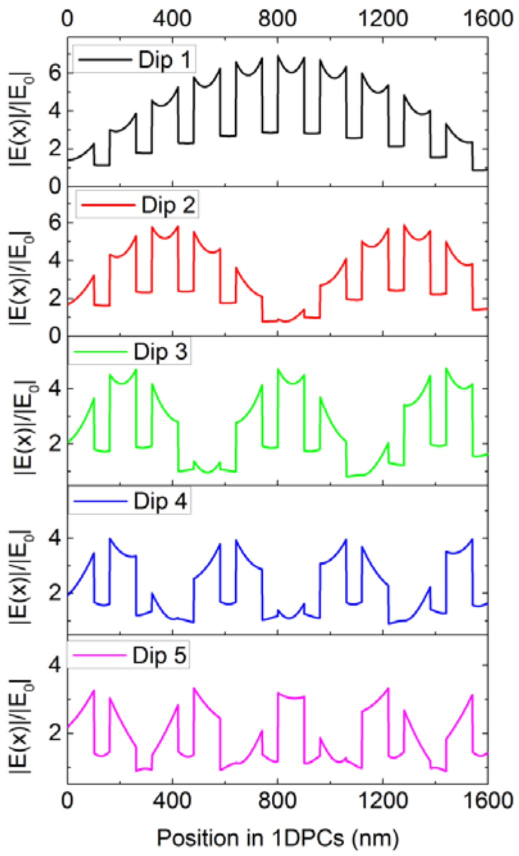

Figure S3 Distribution of the optical electric field in the 1DPCs at Dip 1,2,3,4 and 5. |E(x)| is the modulus of optical electric field in the device and $|E_0|$ denotes that of the incident light

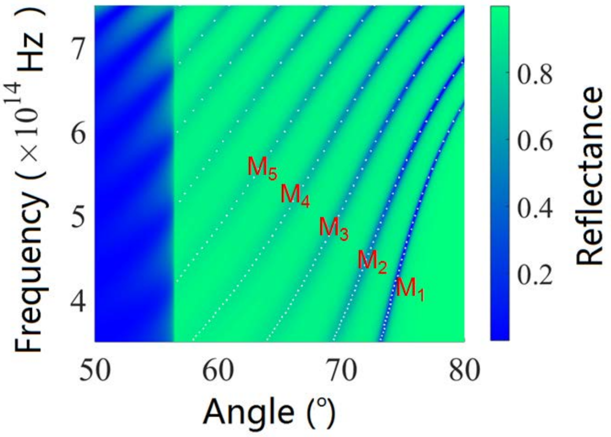

FigureS4 Reflectance as a function of the frequency and incident angle. The white dotted line M*i* (i=1,2,3,4,5) is related to the *i*th-order of the guided mode of the 1DPCs.

Eq. S1 is then used to determine the dispersion relation of the guided mode of the 1DPCs. For this purpose, we calculate the angular reflectance spectra at different frequencies, as is shown in Fig.S4. It is demonstrated that the angular reflectance spectra show minima at the white dotted line. With the incident angle of these reflectance minima as input parameters, $\vec{k}_{gm}$ at a given frequency can be calculated according to Eq. S1. By superposing $\vec{k}_{gm}$ and frequency of the incident light (*f*) onto the dispersion diagram of the 1DPCs, we can obtain dispersion relation of the guided mode of the 1DPCs, as is shown in Fig.S5, where Line M*i* denotes the *i*th-order guided mode of the 1DPCs.

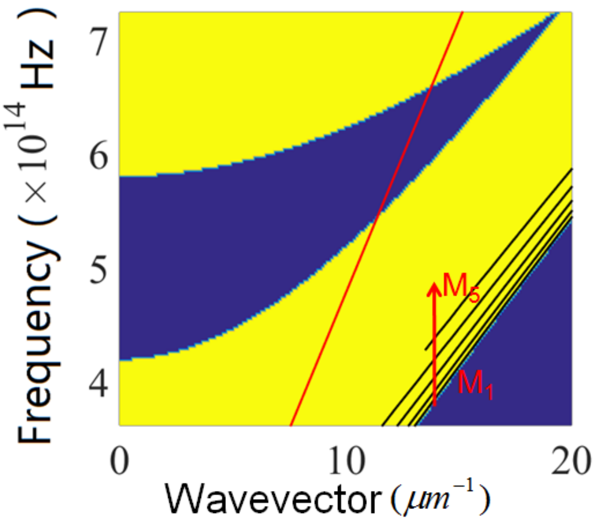

Figure S5 Dispersion diagram of the 1DPCs. The line M*i* (*i*=1,2,3,4,5) denotes the *i*th-order dispersion line of the guided mode of the 1DPCs.The red line denotes the light curve.

References

[S1] Long,Y. Effects of metal electrode reflection and layer thicknesses on the performance of inverted organic solar cells. Sol. Energy Mater. Sol. Cells 94, 744-749 (2010).

[S2] Robertsona, W. M. & May， M. S. Surface electromagnetic wave excitation on one-dimensional photonic band-gap arrays. Appl. Phys. Lett., 74, 29(1999).

[S3] Liscidini, M. & Sipe J. E. Analysis of Bloch-surface-wave assisted diffraction-based biosensors. J. Opt. Soc. Am. B 26, 279 (2009)

[S4] Chen,Y. *et.al.* Study on the propagation mechanism of evanescent waves in one-dimensional periodic photonic crystal. Physics Letters A 379, 2257–2260(2015).
